# Toxicity Assessment and Bioremediation of Deep Eutectic Solvents by *Haloferax mediterranei:* A Step toward Sustainable Circular Chemistry

**DOI:** 10.1002/cssc.202500825

**Published:** 2025-07-08

**Authors:** Guillermo Martínez, Gabriela Guillena, Rosa María Martínez‐Espinosa

**Affiliations:** ^1^ Biochemistry and Molecular Biology and Edaphology and Agricultural Chemistry Department Faculty of Sciences and Multidisciplinary Institute for Environmental Studies (IMEM) University of Alicante Ap. 99 E‐03080 Alicante Spain; ^2^ Organic Chemistry Department and Organic Synthesis Institute (ISO) University of Alicante Ap. 99 E‐03080 Alicante Spain

**Keywords:** biodegradabilities, bioremediations, deep eutectic solvents, ecotoxicities, haloarchaea

## Abstract

Understanding the toxicity of deep eutectic solvents (DESs) remains a major obstacle to large‐scale applications. Existing toxicity studies show inconsistent results due to the choice of different test organisms and methods, synergistic effects between DES components, and their interactions with culture media. This study introduces the haloarchaeon *Haloferax mediterranei* as a novel model to assess both the toxicity and biodegradability of acetylcholine chloride (AcChCl) and choline chloride (ChCl) based DESs. Unlike other models that may not accurately reflect the environmental risks posed by halide‐rich DES residues, *H. mediterranei* is an extremophile naturally adapted to high‐salt and high‐halide environments. DES concentrations of up to 300 mM were well tolerated. AcChCl‐based DESs inhibited growth, likely via medium acidification due to some DES components hydrolysis, ChCl: acetamide has partial effects depending on acetamide concentration, and ChCl: ethylene glycol shows no toxicity. The haloarchaeon metabolizes specific DES components, reducing environmental impact. Urea and AcChCl: urea (100 mM) serve as nitrogen sources, while AcChCl‐based DESs are consumed as carbon sources, likely due to the presence of acetate. *H. mediterranei*'s metabolic versatility and high tolerance to toxic compounds position it as a promising candidate for sustainable bioremediation, advancing circular chemistry, and responsible DES waste management.

## Graphical abstract

The toxicity and biodegradability of deep eutectic solvents (DESs) can be evaluated using *Haloferax mediterranei* as a novel model organism. The haloarchaeon metabolizes specific DES components, highlighting its potential for DES bioremediation. These findings support the use of *H. mediterranei* in sustainable circular chemistry applications.

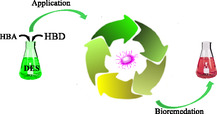

## 1 Introduction

Natural ecosystems worldwide have experienced a dramatic increase in pollution, posing significant challenges to biodiversity, quality of air, water, and soil as well as human health. In this context, legislation and good practices such as using more sustainable processes are crucial tools for removing pollutants and minimizing the production and release of contaminants.^[^ ^1^ ^]^

Furthermore, the chemical industry has witnessed significant advancements in developing solutions that could reduce environmental contamination. The so‐called Green Chemistry aims to create safer and more sustainable chemicals that could replace toxic compounds.^[^ ^2^ ^]^ A notable challenge within the chemical industry has been the use of traditional solvents, which are often toxic and harmful to the environment. However, a promising alternative has recently emerged in the form of deep eutectic solvents (DESs),^[^ ^3^ ^]^ a new class of solvents applicable in various fields such as electrochemistry,^[^ ^4^ ^]^ biocatalysis,^[^ ^5^ ^]^ and extraction.^[^ ^6^ ^]^ These solvents are defined as eutectic mixtures of two or more compounds, with one acting as a hydrogen bond donor (HBD) and the other as a hydrogen bond acceptor (HBA).^[^ ^7^ ^]^ DESs offer numerous advantages over other alternative green solvents, including lower cost, higher biodegradability, lower toxicity, and tunability.^[^ ^8^, ^9^ ^]^

Since their discovery, the toxicity of DESs has been a significant topic of discussion. DESs are often classified as green solvents without the need for rigorous toxicity testing, primarily due to the nontoxic and biodegradable nature of their components.^[^ ^10^ ^]^ However, when a DES is formed, intermolecular interactions, such as hydrogen bonds between HBA and HBD, can alter some properties of the mixture such as viscosity or their toxicity in living beings.^[^ ^11^, ^12^ ^]^ For instance, in some studies monitoring the toxicity of DESs, the interaction through hydrogen bonding between the DES components and the components of the cellular membrane inhibited the bacterial growth. Also, the increased toxicity of DESs compared to their individual components was attributed to charge delocalization enabled by hydrogen bonding or their enhanced ability to delocalize charges.^[^ ^13^ ^]^ Most studies in the field of toxicity have focused on DES mixture toxicity,^[^ ^14^, ^15^ ^]^ without considering the toxicity of each one of their separate components. This approach hampers the evaluation of potential synergistic toxic effects. The toxicity of DESs or their components generally depends on various factors, such as the nature of the HBD and HBA components and their molar ratio. Intrinsic properties of the DESs such as viscosity or density can also influence the toxicological properties.^[^ ^16^ ^]^ Furthermore, changes in the pH of the media, often caused by the hydrolysis of DES components, can affect the survival rate of the organisms. The products of this hydrolysis can interact with the biomolecules present in the medium, altering their properties.^[^ ^17^ ^]^ Different organisms respond differently to the same compound. Therefore, the toxicity or safety of DESs cannot be assumed based only on a single assay using one type of organism as a model.^[^ ^16^ ^]^

In recent years, there has been an increasing interest in the toxicological testing of DESs, as evidenced by the diverse range of life models used in the literature: fish,^[^ ^18^, ^19^, ^20^ ^]^ fungi,^[^ ^18^, ^19^ ^]^ bacteria,^[^ ^15^, ^21^ ^]^ crustaceans,^[^ ^22^ ^]^ or multiple cell lines.^[^ ^23^, ^24^, ^25^ ^]^ Alongside these models, various methodologies have been developed to monitor DES toxicity, each with advantages and drawbacks. The disk diffusion method was reported as the main methodology used: a paper disk impregnated with the DESs is placed on an agar plate, and the toxicity is assessed by measuring the inhibition zone around the disk.^[^ ^26^ ^]^ However, DESs properties such as viscosity, density, and hydrophobicity/hydrophilicity can affect the diffusion of the compound through the agar, influencing the interaction between DESs and the microorganism decreasing and thus reducing the accuracy of the method.^[^ ^12^ ^]^ Other methodologies, like dilution methods, are also used to measure toxicity. Here, the objective is to determine the lowest concentration in which microorganisms can grow.^[^ ^27^ ^]^ However, the presence of large amounts of water can lead to the hydrolysis of DES components, affecting the toxicity of the mixture.^[^ ^12^ ^]^

Although numerous organisms—including bacteria, fungi, and yeast—have been used to test DES toxicity, most of these models thrive in moderate conditions and may not accurately reflect the environmental risks considering that DESs and their residues typically contain high concentrations of halides, which can significantly affect environmental toxicity and waste management. However, extremophilic microorganisms, such as haloarchaea, have never been tested to evaluate DESs toxicity, despite their versatility and potential use for bioremediation strategies to remove pollutants and for biotechnological processes to obtain highly marketed compounds like pigments, enzymes, and bioplastics.^[^ ^28^, ^29^, ^30^ ^]^ Haloarchaea, like *H. mediterranei*, an extremophile naturally adapted to high‐salt and high‐halide environments, provides a more appropriate model for assessing the toxicological impact of DESs in such scenarios due to its metabolic versatility and high tolerance to compounds that are toxic for most living beings.^[^ ^29^ ^]^ Thus, haloarchaea are currently considered model organisms in bioremediation and biotechnology due to the great versatility regarding molecular adaptations, unique metabolisms, and production of biomolecules such as antibiotics or enzymes which are structurally and functionally stable under extreme conditions.^[^ ^31^ ^]^ *H. mediterranei* as a model organism offers several advantages over bacterial counterparts or other haloarchaea species: high growth rates, utilization of various industrial wastewaters as nutrient sources, both aerobic and aerobic metabolism, and high adaptability to simultaneous stress conditions.^[^ ^30^, ^32^ ^]^

Considering the unique metabolic capabilities of haloarchaea, this work uses for the first time a haloarchaeon (*H. mediterranei*) as a model microorganism to monitor the toxicity of these components thus understanding growth kinetics and determining whether this haloarchaeon can survive in the presence of potentially toxic DES. On the other hand, the disposal of DESs‐rich residues as wastewaters could affect natural environments such as hypersaline ones.^[^ ^33^ ^]^ Defined medium, closely resemble these extreme environments, was designed to study the physiological relevance and improve the ecological significance of our findings. Potential uses of this haloarchaeon as a tool for DESs bioremediation are also discussed to shed light on strategies to recycle the significant amount of DESs discarded from large‐scale chemical processes in which those solutions are used.

## 2 Results and Discussion

### 2.1 Analysis of *H.* *Mediterranei* Growth in the Presence of Different DESs

**Figure 1:**
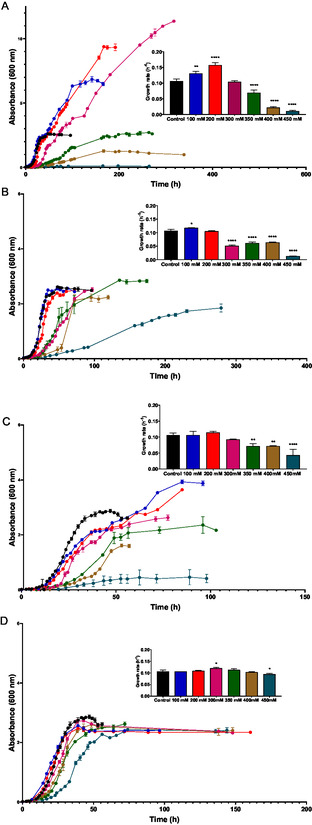
*H. mediterranei* growth curves. Cells were grown in a complex medium with increasing concentrations of A) AcChCl: acetamide‐based DES (1:2), B) ChCl: acetamide‐based DES (1:2), C) AcChCl: urea‐based DES (1:2) and ChCl: ethylene glycol‐based DES (1:2), and D). (Concentrations: Blue = 100 mM; Red = 200 mM; Purple = 300 mM; Green = 350 mM; Brown = 400 mM; Yellow = 450 mM). The growth rate of each concentration was compared to the control culture to evaluate statistical significance. *P*‐value ≤ 0.05 (*), *p*‐value ≤ 0.01 (**), *p*‐value ≤ 0.001 (***), *p*‐value ≤ 0.0001 (****).

To assess *H. mediterranei* tolerance to these DESs, cells [strain R‐4 (ATCC33500)] were grown in complex media buffered with (3‐(*N‐*morpholino)propanesulfonic acid) (MOPS) and supplemented with increasing concentrations of four different DESs based on AcChCl or ChCl, upon 450 mM (see Supporting Information for details). The growth curves for *H. mediterranei* in the presence of DESs (100–450 mM) and individual components (100–450 mM and 200–900 mM for HBA and HBD, respectively), as well as the growth rates, are shown in **Figure** **1**. The concentration selection was based on previous studies where the toxicity of AcChCl: acetamide‐based DESs was tested with *Escherichia coli* as model organisms,^[^ ^17^ ^]^ where DES were well tolerated up to a 450 mM concentration.

The control culture exhibited the expected growth profile, characterized by a short lag phase, a brief exponential phase, and finally, a stationary phase with a maximum optical density (OD_600_) of ≈2.5. In the presence of AcChCl: acetamide‐based DESs (Figure 1A), the lag phase duration varied depending on the DES concentration. The increase in concentration led to a prolonged lag phase, as well as a lower growth rate. Interestingly, DES concentrations below 200 mM enhanced cell growth, reaching a maximum optical density of 11 in the stationary phase. *H. mediterranei* tolerated concentrations up to 350 mM, achieving an OD_600_ in the stationary phase which is similar to the OD_600_ observed in the control culture. At 400 mM, the OD_600_ dropped to 1, and concentrations above 450 mM completely inhibited cell growth. Growth curves for DES concentrations between 100 and 350 mM displayed a characteristic diauxic growth, with two distinct growth phases separated by a short lag phase. Figure 1B summarizes the growth of the microorganism in the presence of ChCl: acetamide‐based DES. Higher concentrations of DES extended the lag phase. Lower concentrations, specifically 200 mM, resulted in a maximum OD_600_ similar to that of the control culture. Growth was significantly impacted at concentrations above 300 mM, which, although tolerated, dramatically decreased the growth rates.

Cultures with AcChCl: urea‐based DES exhibited intermediate behavior compared to the two previous DESs. Cells grown with 100 and 200 mM of DES showed diauxic growth, reaching a maximum OD_600_ of 4 (Figure 1C). As described for the previous DESs, an increase in the concentration extended the lag phase. Concentrations below 300 mM did not affect the growth of the microorganism, while 450 mM inhibited growth, with an OD_600_ of 0.5 in the stationary phase.

Finally, Figure 1D displayed the growth of the haloarchaea in the presence of ChCl: ethylene glycol‐based DES. No differences in growth were observed with increasing DES concentration. The DES did not affect growth, and a maximum OD_600_ similar to the control culture was achieved across all the concentrations.

ChCl‐based DESs toxicities have been extensively studied in the literature using different methodologies. Hayyan and co‐workers^[^ ^21^ ^]^ initially employed the disk diffusion method to investigate the toxicity of a mixture between ChCl and ethylene glycol (1:3), finding no toxic effects on different bacteria. Consistent results were observed in the present study, where no significant impact on the growth of haloarchaea was noted in the presence of a similar ChCl: ethylene glycol mixture (1:2). Low toxicity was reported in a study where ChCl: acetamide (1:2) and ChCl: ethylene glycol (1:2) were tested against different bacteria (gram positive and gram negative).^[^ ^11^ ^]^ The low toxicity caused by ChCl: ethylene glycol (1:2) in these bacteria using disk diffusion methods aligns with the findings observed with *H. mediterranei,* suggesting the green character of this mixture.

In contrast, results obtained with ChCl: acetamide (1:2) indicated a more pronounced impact on the haloarchaea, as the highest concentration (450 mM) significantly inhibited the growth of the microorganism. The broth dilution method has been used to determine the minimal concentration at which an antimicrobial agent inhibits growth.^[^ ^12^ ^]^ Using this method, ChCl: acetamide (1:2) and ChCl: ethylene glycol (1:1) were tested to ascertain their EC_50_ values against *E. coli*, revealing concentrations of 275.2 and 434.4 mM, respectively, which induced a biological response at half of the maximum observed response.^[^ ^13^ ^]^ In agreement with that study, low concentrations of these DESs did not affect the growth of haloarchaea. However, increasing the concentration of ChCl: acetamide (1:2) led to a marked inhibitory effect, with a growth rate reduction of nearly 90% at 450 mM. This is comparable to the results obtained with *E. coli*, where inhibition levels exceeded 72.8%. Notably, while 400 mM of this DES inhibited *E. coli* growth by ≈80%, *H. mediterranei* showed no significant impact up to 450 mM. Furthermore, the toxicity of ChCl: ethylene glycol (1:2) was also evaluated using colorimetric assays. Low impact of this DESs, consistent with our study, was caused on *Saccharomyces cerevisiae* growth, with cell viability remaining close to 100%.^[^ ^34^ ^]^ Significantly different results were obtained with *Arthrobacter simplex and Bacillus cereus,* where ChCl:ethylene glycol‐based DESs showed relative decreasing the membrane integrity by ≈50% in media containing around 13% v/v of the solvent.^[^ ^35^, ^36^ ^]^

The observed differences in DES toxicity are primarily attributed to the use of different model microorganisms and methodologies. To specifically assess the influence of the HBA or HBD without the bias introduced by differences in microbial tolerance, standardized toxicity assays using the same model organism and controlled conditions are essential. Using the methodology that is described in this current study, *H. mediterranei* showed no growth inhibition in the presence of concentrations of ChCl: ethylene glycol up to 450 mM, while it was affected by 450 mM of ChCl: acetamide as previously described.

DESs based on AcChCl showed inhibition of the growth of haloarchaea. Two main hypotheses were considered: the toxicity caused by the DES itself or toxicity resulting from changes in the culture medium properties induced by these compounds. Parameters such as pH can significantly influence the survival rate of the microorganisms in their environment. DESs or their components can interact with the medium and alter its physicochemical properties.^[^ ^17^ ^]^ In this case, the potential hydrolysis of DES components was investigated by monitoring pH changes throughout the culture period (see Supporting Information). For all the cultures containing DESs or individual components, starting (after inoculation of microorganism and compounds) was set to 7.40. AcChCl‐based DESs showed a dramatic drop in pH, significantly higher than the observed in ChCl‐based ones. Specifically, in AcChCl: acetamide DES, the reduction in pH was found to correlate with the concentration of the DES. At 100 mM, the pH was ≈7.01, whereas at 450 mM it dropped to 5.77. Notably, at concentrations above 200 mM, the pH reduction became particularly pronounced, with no recovery observed throughout the culture period. A similar trend was observed for AcChCl: urea‐based DESs, where the pH decreased to 5.34 at 450 mM, and no pH recovery was noted at concentrations above 300 mM. In both cases, the 100 mM MOPS buffer was insufficient to counteract the acidification caused by these compounds. Previous studies have reported similar pH drops in AcChCl: acetamide (1:2)‐based DES in Luria–Bertani (LB) medium buffered with 100 mM tris(hydroxymethyl)aminomethane hydrochloric acid (Tris)‐ (HCl).^[^ ^17^ ^]^ In contrast, the ChCl‐based DESs (ChCl: acetamide and ChCl: ethylene glycol) did not cause a pH drop; rather, an increase in pH was observed during the culture. Finally, experiments with the individual components revealed that neither acetamide nor urea alone altered the pH during the culture. However, increasing concentrations of AcChCl alone led to a pH drop, reaching 5.50 at 400 mM after 24 h, though in these cultures the pH gradually recovered over time.

These results suggest that the observed acidification is primarily due to the hydrolysis of acetylcholine chloride. Changes in the structure of the DESs due to interactions with high concentrations of water have been described in the literature. Kumari and co‐workers^[^ ^37^ ^]^ studied the influence of water content on the structure of ChCl: urea‐based DESs, finding that water can act as a second hydrogen bond donor or acceptor, solvating chloride anions and ammonium cations. This addition of water significantly affects the properties and behavior of the DESs.^[^ ^38^ ^]^ Hydrolysis of DESs can occur in a liquid medium, potentially leading to the formation of new compounds that interact with the medium and alter its physicochemical properties.^[^ ^12^, ^17^ ^]^ In this current study, AcChCl may undergo hydrolysis, producing components such as choline and acetic acid. The presence of acetic acid could explain the observed acidification of the medium.

### 2.2 Potential Synergistic Effect between Components of DESs

**Figure 2:**
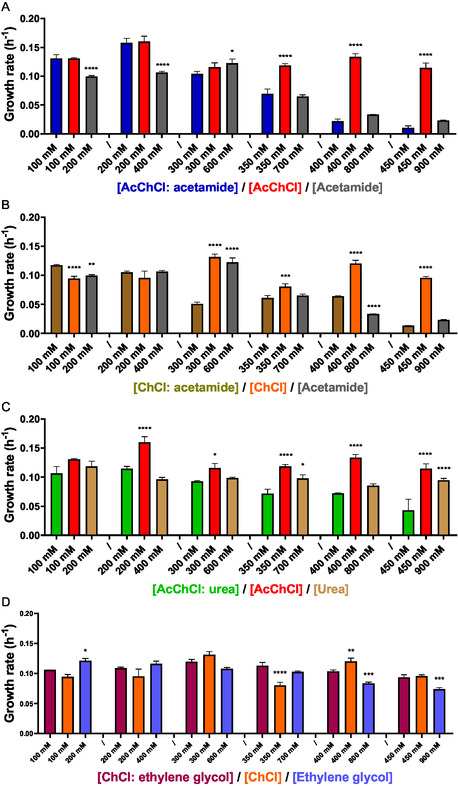
Specific growth rate of *H. mediterranei* during the exponential phase in the presence of increasing concentrations of A) AcChCl: acetamide‐based DES and individual components, B) ChCl: acetamide‐based DES and individual components, C) AcChCl: urea‐based DES and individual components, and D) ChCl: ethylene glycol‐based DES and individual components. The growth rate of individual components was compared to the corresponding concentration of its DES to evaluate statistical significance. *P*‐value ≤ 0.05 (*), *p*‐value ≤ 0.01 (**), *p*‐value ≤ 0.001 (***), *p*‐value ≤ 0.0001 (****).

Experiments were performed to study the potential synergistic effect of the DES formation. Cultures were grown in a buffered complex medium supplemented with HBAs/HBDs individually at concentrations equivalent to those of the DESs (100–450 mM of HBA and 200–900 mM of HBD). **Figure** **2** illustrates the growth rates obtained in culture media with different DESs/HBAs/HBDs by comparing each concentration of the DES with the corresponding concentration of the individual compound present in the DES.

In the study involving AcChCl: acetamide‐based DES and its components (Figure 2A), the presence of the DES caused greater decrease in growth as the concentration increased compared to AcChCl. At low concentrations (100–300 mM), DES and AcChCl did not show significant differences in growth. However, at concentrations higher than 300 mM, the decrease in growth in the presence of DES was similar to the inhibition caused by acetamide, while growth with AcChCl remained constant along all the concentrations.

A similar behavior is observed in Figure 2B, where the growth of the haloarchaea was affected when increasing the concentration of ChCl: acetamide (1:2) and acetamide. Conversely, no growth inhibition was observed with increasing concentrations of ChCl in the culture. Additionally, there was no significant drop in the medium with these components during the culture (see Supplementary data). In this case, it seems that there is a correlation between the concentration of acetamide and the toxicity.

As previously mentioned, the AcChCl: urea‐based DES showed notable growth inhibition at 450 mM, with a maximum OD_600_ of 0.5. At this concentration, the pH decreased to 5.34 after 96 h of culture. In terms of its components, the pH decreased to 6.45 with 450 mM AcChCl and increased to 7.77 with 900 mM of urea (see Supplementary Information). Although the medium contained the same concentration of both DES and AcChCl, a more pronounced decrease in pH was recorded in the presence of the DES. Studying the different growth rates in culture media, a clear synergistic effect is evident in Figure 2C. The growth rate is inhibited in the presence of increasing concentrations of the DES, while remains constant in the presence of individual components across all tested concentrations.

No synergistic effect was observed between ChCl and ethylene glycol in the formation of the DES. After 96 h of culture, no decrease in pH was observed with any of the components (see Supporting Information). Increasing the concentration of all the components did not significantly inhibit growth, and no difference was observed between the DES and the individual components (Figure 2D).

The synergistic effects among the components of DESs have not been thoroughly investigated in most studies addressing DES toxicity. Only a limited number of studies employing disk diffusion methods have evaluated the toxicity of individual components alongside that of the DESs themselves. The toxicity of the individual components corroborates previous findings, wherein ChCl exhibited no toxic effects against *E. coli*.^[^ ^39^ ^]^ Moreover, the inclusion of ChCl in various DESs with different HBDs did not enhance the toxicity of the mixture, indicating an absence of synergistic effects. Notably, a mixture comprising ChCl and acetamide, which demonstrated a synergistic effect in our investigation, was not examined in prior studies. In research involving *A. simplex*, the evaluation of membrane integrity revealed significant discrepancies when contrasting the individual effects of ChCl and ethylene glycol with their corresponding DES. These results diverge from those reported in the current study. Although higher inhibition zones were recorded with the individual components relative to the DES, *H. mediterranei* did not exhibit this trend against either the DES or the individual components. ChCl resulted in partial inhibition of the microorganism, while urea had no discernible effect on its growth.^[^ ^40^ ^]^ In our study, ChCl and ethylene glycol did not exhibit toxic effects on *H. mediterranei*, and no synergistic effect was observed following the formation of the DES. Notably, discrepancies can be observed in the study conducted by Torregrosa‐Crespo^[^ ^17^ ^]^ and colleagues, which reported that AcChCl: acetamide‐based DES exhibited greater toxicity compared to acetamide and AcChCl when assessed individually. This supports the notion of heightened toxicity associated with the AcChCl: acetamide‐based DES in comparison to AcChCl alone. While that study suggested that the toxicity of the DES surpassed that of acetamide, our findings imply a significant relationship between these two components, with the growth rates of the haloarchaeon diminishing in the presence of this DES, akin to the effects observed with increasing concentrations of acetamide.

### 2.3 Potential Consumption of DES and Individual Components as a Nitrogen Source

In the literature, DESs are generally considered biodegradable mixtures due to the nature of their components. However, biodegradation tests are still limited, and further studies are needed to thoroughly assess this biodegradation. To investigate biodegradation (degradation, transformation, or accumulation inside the cell) or assimilation (use of DESs or individual components as a source of nutrients), a buffered minimal medium was supplemented with increasing concentrations of DESs and their separate components.

Two different control cultures were prepared to study the growth in an optimal culture medium: one with NH_4_Cl as the nitrogen source (control +N) and one without any nitrogen source (control –N). In the presence of nitrogen, a standard microbial growth curve was observed, with a rapid exponential phase and a maximum OD_600_ of 3.5 at the stationary phase. In contrast, the negative control showed minimal growth, with a maximum OD_600_ of 0.3, potentially due to residual nitrogen from the preinoculum prepared in an optimal medium.

**Figure 3:**
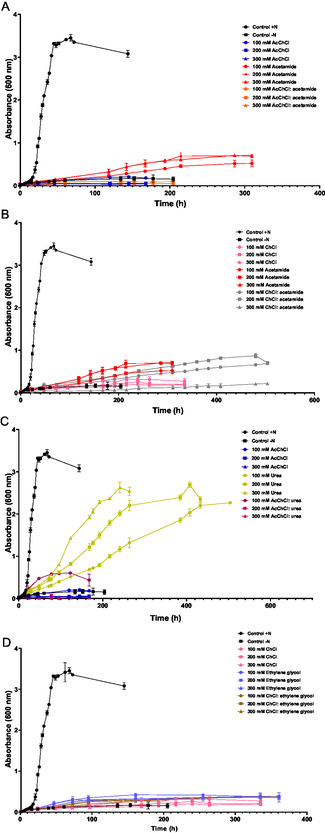
Growth curves of *H. mediterranei* in a minimal medium with or without a nitrogen source, supplemented with increasing concentrations of A) AcChCl: acetamide‐based DES and individual components, B) ChCl: acetamide‐based DES and individual components, C) AcChCl: urea‐based DES and individual components, and D) ChCl: ethylene glycol‐based DES and individual components.

In this study, two different trends were observed. Cultures containing AcChCl: acetamide‐based DESs (**Figure** **3**A) and both ChCl‐based DESs (Figure 3B,D) and their individual components did not show significant growth, with a maximum OD_600_ of 0.5. In contrast, a significant difference was observed with AcChCl: urea‐based DES. Although, growth was slower than in the control culture with NH_4_Cl, a maximum OD_600_ of 2.7 was achieved in cultures with urea as a nitrogen source. The higher the concentration of urea, the faster the growth of the microorganism (Figure 3C). Lower concentrations of AcChCl: urea enables growth until a maximum OD_600_ of 0.6.

*H. mediterranei* represents one of the most extensively studied model organisms regarding nitrogen metabolism. This haloarchaeon can utilize different nitrogen sources such as nitrite, nitrate, or ammonium under aerobic conditions.^[^ ^41^ ^]^ All metalloenzymes involved in denitrification (the reduction of nitrate to dinitrogen) are also present in this microorganism.^[^ ^42^ ^]^

However, research related to the use of urea as a nitrogen source is still limited. Mizuki and co‐workers^[^ ^43^ ^]^ searched for urease activity in several strains belonging to extreme halophiles, including *H. mediterranei* ATC33500. They screened for urease activity using a urea‐phenol red‐agar medium and measured the activity using the phenol–hypochlorite method. Among the haloarchaea species tested, only a few from the genus *Haloarcula* exhibited urease activity, with *Haloarcula hispanica* showing the highest activity.

*H. mediterranei* did not display any urease activity, although homologous genes to the urease genes of species of *Haloarcula* were detected using primers that amplified these genes in *Haloarcula* *spp*.^[^ ^43^ ^]^ Another study by Hashemzahi and coworkers^[^ ^44^ ^]^ examined enzyme production by different microorganisms inhabiting the shore of the Oman Sea. They identified six different strains of the genus *Haloferax* in this ecosystem. Using a urea–phenol red‐agar medium, they found that among 47 different strains, there were weak (16 strains), moderate (15 strains) and strong urease producers (16 strains), although they did not specify which strains fell into each category. In this study, *Halococcus saccharolyticus* was identified as the major urease producer.

### 2.4 Potential Consumption of DES and Individual Components as a Carbon Source

**Figure 4:**
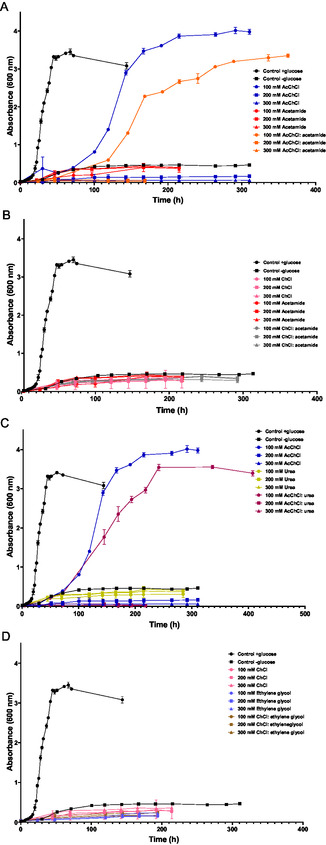
Growth curves of *H. mediterranei* in minimal medium supplemented with or without carbon source and increasing concentrations of A) AcChCl: acetamide‐based DESs and individual components, B) ChCl: acetamide‐based DESs and individual components, C) AcChCl: urea‐based DESs and individual components, and D) ChCl: ethylene glycol‐based DESs and individual components.

To study whether DES or their separate components could serve as carbon sources for *H. mediterranei*, a buffered minimal medium without glucose was supplemented with these components at concentrations ranging from 100 to 300 mM. Control cultures were also prepared with and without glucose to establish growth under optimal and deficient conditions, respectively. **Figure** **4** shows the growth curves of *H. mediterranei* in a minimal medium where DESs and their separate components were provided as carbon sources.

In the control culture with glucose, a short lag phase was followed by a rapid exponential phase, reaching a stationary phase with an OD_600_ of 3.4. Conversely, cultures in a minimal medium without glucose reached an OD_600_ of 0.4, likely due to the residual glucose from the preinoculum. Cultures grown with ChCl‐based DESs (Figure 4B,D) and their components (ChCl, acetamide, and ethylene glycol), showed no significant growth, as these compounds could not serve as carbon sources in the absence of glucose. All the growth curves were lower than the control culture without glucose.

Regarding the growth in the presence of urea as individual component, no growth was identified during the culture.

However, at the lowest concentrations of AcChCl: acetamide, AcChCl: urea and AcChCl, the cultures exhibited similar growth patterns. In the presence of 100 mM of these components, the growth curve displayed significantly longer lag phases compared to the minimal medium with glucose.

The rapid exponential phase began after 70 h of culture, with the stationary phase reaching a maximum OD_600_ of 4 with AcChCl (Figure 4A or C), 3.3 with AcChCl: acetamide (Figure 4A) and 3.5 with AcChCl: urea (Figure 4C).

As previously described, hydrolysis of the components in AcChCl‐based DESs can increase acetate concentration in the medium. The use of acetate as a carbon source has been studied in the haloarchaea *Haloferax volcanii*. Acetate metabolism involves two different stages: (i) uptake of the acetate into the cell and (ii) activation of acetate to acetyl‐CoA, which is metabolized in the catabolism of CO_2_ via enzymes of the citric acid and respiratory chain.^[^ ^45^ ^]^

Acetic acid (pK_a_ = 4.76) is likely present in the culture media (pH near 6) as acetate, with uptake coordinated by a specific transport system.^[^ ^46^ ^]^ In the case of *H. mediterranei*, different studies have confirmed the use of acetate as a carbon source. Nearly three decades ago, Oren and Gurevich^[^ ^47^ ^]^ observed that *H. mediterranei* could grow and exhibit high isocitrate lyase activity (an enzyme in the glyoxylate cycle) when grown in a medium with acetate as the carbon source. In a biotechnological context, acetate derived from food waste has been used as a carbon source to produce polyhydroxyalkanoates.^[^ ^48^ ^]^ Fang and co‐workers^[^ ^49^ ^]^ studied the influence of using sodium acetate as a carbon source in the production of C_50_ carotenoids in *H. mediterranei*. They found that replacing glucose with acetate significantly increased carotenoid production, demonstrating the efficacy of acetate as a carbon source in the culture.

Recently, Mitra and co‐workers identified the genes in charge of the activation of acetate into acetyl‐coA in the haloarchaeon *H. mediterranei,* confirming the ability to consume acetate as a carbon source in this strain.^[^ ^50^ ^]^

## 3 Conclusion

The present study was designed to determine the effect of different DESs on the growth of the haloarchaeon *H. mediterranei*, revealing different behaviors across the used components. AcChCl‐based DESs demonstrated increased toxicity at higher concentrations, primarily due to the extreme acidification of the culture medium caused by the potential hydrolysis of the DESs. However, low and intermediate concentrations (100–300 mM) did not exhibit toxicity; instead, they increased growth rates and a maximum OD_600_ of 11.4 was observed in the stationary phase. For ChCl: acetamide‐based DESs no growth increase was observed at low concentrations, and higher concentrations partially inhibited growth despite stable pH levels during culture. Regarding ChCl: ethylene glycol, showed neither inhibition nor enhancement of growth at any tested concentration. These findings suggest that DESs toxicity is significantly influenced by the HBA/HBD in the mixture and their potential interactions with the culture media.

Depending on the interaction between HBA and HBD, the toxicity of the mixture can vary considerably. This study found that the toxicity of ChCl: acetamide‐based DES was primarily associated with the presence of acetamide in the mixture. Further research should investigate whether the observed toxicity at high concentrations of these DESs is due to acidification, acetamide toxicity, or both. AcChCl: urea‐based DES showed a potential synergistic effect, as growth decreased with increasing concentrations, while their components did not inhibit growth. The study of ChCl: ethylene glycol reveals that both the DES and its components had no significant impact on the growth of the microorganism.

This project was undertaken to design potential bioremediation strategies for these compounds and evaluate their potential use as nitrogen and carbon sources. This study is the first to report on the use of urea as a nitrogen source by *H. mediterranei*. Increasing urea concentrations led to a higher maximum OD_600_ of 2.69 in the stationary phase. Although low OD_600_ (0.6) was observed at low concentrations of AcChCl: urea, the potential for bioremediation can be optimized. Future research should focus on the consumption of urea by *H. mediterranei*, as no urease‐related genes have been identified in this microorganism before.

One significant finding from this study is the potential for bioremediation strategies using these compounds as carbon sources. The results suggest that low concentrations of AcChCl‐based DESs can be used as a carbon source. Despite increased lag and exponential phases, higher OD_600_ at the stationary phase was achieved under these conditions. Future work should analyze the potential use of low concentrations of AcChCl: urea‐based DES as both nitrogen and carbon sources, given that the haloarchaea can utilize these compounds separately.

The results underscore the importance of considering both the individual components and their interactions within DES mixtures when evaluating their toxicity. The observed acidification and subsequent growth inhibition highlight the need for further research to elucidate the mechanisms underlying the hydrolysis of DESs and their impact on microbial growth. Understanding these interactions can inform the design of less toxic, more environmentally friendly solvents by selecting components that minimize negative synergistic effects. Further studies should investigate the long‐term effects of DES exposure on *H. mediterranei*, including potential adaptive responses at the molecular level. Additionally, exploring the applicability of these findings to other extremophiles could expand the recyclability of DESs thus being used as C or N sources for microbial growth in various biotechnological processes aiming at the production of marketed biomolecules contributing to circular chemistry processes.

## 4 Experimental Section

4.1

4.1.1

##### Chemicals and Materials

All chemicals and reagents used in this study were of analytical grade and used without further purification. Acetylcholine chloride (99%, Thermo Scientific), acetamide (99%, Alfa Aesar), choline chloride (>98%, Thermo Scientific), urea (>99%, Sigma‐Aldrich), ethylene glycol (99%, Alfa Aesar), MOPS buffer (99%, Thermo Scientific), NaCl (>99%, PanReac AppliChem), MgSO_4_·7 H_2_O (>99%, PanReac AppliChem), MgCl_2_·6 H_2_O (>98%, PanReac AppliChem), KCl (>99.5%, PanReac AppliChem), NaHCO_3_ (>99.7%, PanReac AppliChem), NaBr (>99%, PanReac AppliChem), CaCl_2_·2 H_2_O (>99%, VWR), NH_4_Cl (99.5%, PanReac AppliChem), NaH_2_PO_4_·2 H_2_O (>99%, PanReac AppliChem), Na_2_HPO_4_·12 H_2_O (>98.5%, PanReac AppliChem), FeCl_3_ (>98%, Sigma‐Aldrich), and glucose (99%, Alfa Aesar) were obtained from commercial suppliers. Yeast extract was purchased from Condalab.

##### Synthesis of DESs

Four different DESs were synthesized (**Table** **1**) by combining known molar masses of choline chloride and acetamide or ethylene glycol and acetylcholine chloride and acetamide or urea. The 2:1 HBD: HBA molar ratio was selected because it corresponded to the eutectic composition for all these systems.

**Table 1:** Combination of the different individual compounds, their proportion in the DES, and the density of the DES.

DESs	HBD	HBA	Molar ratio	Density [g/cm^3^]
**DES 1**	Acetamide	Acetylcholine chloride	2:1	1.090^[^ ^54^ ^]^
**DES 2**	Acetamide	Choline chloride	2:1	1.085^[^ ^11^ ^]^
**DES 3**	Urea	Acetylcholine chloride	2:1	1.206^[^ ^55^ ^]^
**DES 4**	Ethylene glycol	Choline chloride	2:1	1.114^[^ ^11^ ^]^

Approximately 300 g of each DESs were prepared by mixing the individual components in the appropriate molar ratio stirring the mixture at 300 rpm and 90 °C for 2 h until the solid powder turned into a liquid without any suspensions. This process was carried out under argon atmosphere since DESs were hygroscopic compounds to control the water present in the mixture.^[^ ^51^ ^]^ DESs were allowed to cool down at room temperature and saved into a falcon until the inoculation in the culture media. DES1 solidified at room temperature, so before introducing it into the culture medium, it was heated at 80 °C to form a liquid mixture.

##### Microbial Strain and Culture Media

The haloarchaeon H. *mediterranei* strain R‐4 (ATCC33500) was used to monitor the toxicity of 4 different DESs, as well as the individual components of DES to study a potential synergistic effect. The 4 DESs as well as their individual components were also used as nitrogen and carbon sources to monitor the metabolic capability of *H. mediterranei* to biodegrade/bioassimilate those compounds.

To monitor the toxicity of DESs and individual components, cells were grown in a complex medium containing 25% of inorganic salts solution (per liter): sodium chloride (NaCl), 234 g; magnesium chloride hexahydrate (MgCl_2_·6H20), 41.5 g; magnesium sulfate heptahydrate (MgSO_4_·7H_2_0), 59.3 g; calcium chloride dihydrate (CaCl_2_·6H_2_0), 1.457 g; potassium chloride (KCl), 6 g; sodium bicarbonate (NaHCO_3_), 0.2 g; sodium bromide (NaBr), 0.7 g^[^ ^52^ ^]^ and 0.5% w/v yeast extract. MOPS was added to the culture media to keep the pH of the culture due to possible acidification of the medium by the addition and/or subsequential modification of DES due to biotic and abiotic reactions. The pH of the medium was adjusted to 7.3 and monitored during the first 96 h of incubation. Cultures were sterilized by autoclave (Autoclave Presoclave III 80 L, JP Selecta) at 121 °C for 21 min. After sterilization, the media were supplemented with concentrations of DESs from 0 (control) to 450 mM. The possible synergistic effect in the DES was also evaluated by adding the individual components separately. The concentrations of the HBA and HBD used corresponded to those contained in the DES (HBD is two times higher). Both DES and individual components were sterilized by UV for 30 min and then added to the sterilized medium. The incubation experiments were conducted in 500 mL Erlenmeyers containing 100 mL of complex medium and inoculated with 1% v/v of *H. mediterranei* cells. This preinoculum was grown in the same complex medium containing 25% of inorganic salts solution and 0.5% w/v yeast extract, buffered with 100 mM of MOPS. Cells were grown at 42 °C and constant shaking at 170 rpm (Multitron Standard, Infors HT). Preinoculum was obtained in the exponential phase to obtain more metabolically active cells. Growth conditions also included 42 °C and constant shaking at 170 rpm. Haloarchaeon growth was monitored by measuring the absorbance at 600 nm (Cary 60 UV–vis spectrophotometer, Agilent Technologies). To obtain a well‐defined growth curve, all studies were performed with 6 replicates. Growth rate (*μ*) and doubling time (d.t) were calculated according using the equations: ***μ*** **= ln(** ***X*** **‐** ***X***_**0**_ **)/(** ***t*** **‐** ***t***_**0**_ **)**, where *X* and *X*_o_ represent the absorbance value at the end and the start of the exponential phase, respectively, and *t* and *t*_0_ the time in this period.^[^ ^53^ ^]^

To monitor the growth using DESs and individual components as nitrogen and carbon sources (in connection with potential bioremediation capabilities), cells were grown in a defined minimal medium containing 25% w/v of inorganic salts as previously mentioned, 20% glucose, 15 mM NH_4_Cl, 1 mM phosphate salts (sodium dihydrogen phosphate dihydrate/disodium hydrogen phosphate dodecahydrate) (Na_2_HPO_4_/NaH_2_PO_4_), and 0.005 g/L of ferric chloride (FeCl_3_). In the cultures for the study of the potential consumption of DESs or individual components as nitrogen and carbon source, NH_4_Cl or glucose was not added, respectively (DESs or individual components were added in substitution). DESs were tested in 3 different concentrations: 100, 200, and 300 mM. HBAs were introduced in the same concentrations as DESs, while HBDs were introduced two times higher (200, 400, and 600 mM). To monitor the growth, the absorbance value at 600 nm was measured during the culture. Cultures were carried out in triplicates.

## Conflict of Interest

The authors declare no conflict of interest.

## Supporting information

Supplementary Material

## Data Availability

The data that support the findings of this study are openly available in [RUA. Repositorio Institucional de la Universidad de Alicante] at [http://hdl.handle.net/10045/155114], reference number [0].
