# Supplementary material for: Toxicity Assessment and Bioremediation of Deep Eutectic Solvents by Haloferax mediterranei: A Step toward Sustainable Circular Chemistry
Source: ChemSusChem. 2025 Jul 8;18(16):e202500825. doi: 10.1002/cssc.202500825 (PMC12330313; doi:10.1002/cssc.202500825)
Supplement: Supplementary file 1 — Supplementary Material [file CSSC-18-e202500825-s001.pdf]

Toxicity Assessment and Bioremediation of Deep Eutectic Solvents by *Haloferax mediterranei*: A Step Toward Sustainable Circular Chemistry

Guillermo Martínez, [a] Gabriela Guillena, [b]* Rosa María Martínez-Espinosa [a]*

[a] Biochemistry and Molecular Biology and Edaphology and Agricultural Chemistry Department, Faculty of Sciences and Multidisciplinary Institute for Environmental Studies (IMEM). University of Alicante Ap. 99, E-03080 Alicante, Spain

[b] Organic Chemistry Department and Organic Synthesis Institute (ISO). University of Alicante. Ap. 99, E-03080 Alicante, Spain

Table of contents

Experimental section 1

Chemicals and materials 1

Synthesis of DESs 1

Microbial strain and culture media 2

Statistical analysis 3

pH measurements data 3

Table S2. pH measurements during first 96 hours of culture for all the DESs and individual components tested in complex media. pH measures were carried out using MU 6199L multi-parameter (pHenomenal, VWR). 3

References 5

Experimental section

Chemicals and materials

All chemicals and reagents used in this study were of analytical grade and used without further purification. Acetylcholine chloride (99%, Thermo Scientific), acetamide (99%, Alfa Aesar), choline chloride (>98%, Thermo Scientific), urea (>99%, Sigma-Aldrich), ethylene glycol (99%, Alfa Aesar), MOPS buffer (99%, Thermo Scientific), NaCl (>99%, PanReac AppliChem), $MgSO_4 \cdot 7H_2O$ (>99%, PanReac AppliChem), $MgCl_2 \cdot 6H_2O$ (>98%, PanReac AppliChem), KCl (>99.5%, PanReac AppliChem), $NaHCO_3$ (>99.7%, PanReac AppliChem), NaBr (>99%, PanReac AppliChem), $CaCl_2 \cdot 2H_2O$ (>99%, VWR), NH_4Cl (99.5%, PanReac AppliChem), $NaH_2PO_4 \cdot 2H_2O$ (>99%, PanReac AppliChem), $Na_2HPO_4 \cdot 12H_2O$ (>98.5%, PanReac AppliChem), $FeCl_3$ (>98%, Sigma-Aldrich), and glucose (99%, Alfa Aesar) were obtained from commercial suppliers. Yeast extract was purchased from Condalab.

Synthesis of DESs

Four different DESs were synthesized (Table S1) by combining known molar masses of choline chloride and acetamide or ethylene glycol, and acetylcholine chloride and acetamide or urea. The 2:1 HBD: HBA molar ratio was selected because it corresponds to the eutectic composition for all these systems.

Table S1. Combination of the different individual compounds, their proportion in the DES and the density of the DES.

DESs	HBD	HBA	Molar ratio	Density (g/ 〖cm^3))
**DES 1**	Acetamide	Acetylcholine chloride	2:1	1.090[1]
**DES 2**	Acetamide	Choline chloride	2:1	1.085[2]
**DES 3**	Urea	Acetylcholine chloride	2:1	1.206[3]
**DES 4**	Ethylene glycol	Choline chloride	2:1	1.114

300 grames of each DESs were prepared by mixing the individual components in the appropriate molar ratio stirring the mixture at 300 rpm and 90ºC for 2 hours until the solid powder turned into a liquid without any suspensions. This process was carried out under argon atmosphere since DESs are hygroscopic compounds to control the water present in the mixture.[49] DESs were allowed to cool down at room temperature and saved into a falcon until the inoculation in the culture media. DES1 solidified at room temperature, so before introducing it into the culture medium, it was heated at 80ºC to form a liquid mixture.

Microbial strain and culture media

The haloarchaeon *Haloferax mediterranei* strain R-4 (ATCC33500) was used to monitor the toxicity of 4 different DESs, as well as the individual components of DES to study a potential synergistic effect. The 4 DESs as well as their individual components were also used as nitrogen and carbon sources to monitor the metabolic capability of *H. mediterranei* to biodegrade/bioassimilate those compounds.

To monitor the toxicity of DESs and individual components, cells were grown in a complex medium containing 25% of inorganic salts solution (per litre): sodium chloride (NaCl), 234 g; magnesium chloride hexahydrate ($MgCl_2\cdot 6H20$), 41.5 g; magnesium sulfate heptahydrate ($MgSO_4\cdot 7H_20$), 59.3 g; calcium chloride dihydrate ($CaCl_2\cdot 6H_20$), 1.457 g; potassium chloride (KCl), 6 g; sodium bicarbonate ($NaHCO_3$), 0.2 g; sodium bromide (NaBr), 0.7 g[50] and 0.5% (w/v) yeast extract. MOPS was added to the culture media to keep the pH of the culture due to possible acidification of the medium by the addition and/or subsequential modification of DES due to biotic and abiotic reactions. The pH of the medium was adjusted to 7.3 and monitored during the first 96 hours of incubation. Cultures were sterilised by autoclave (Autoclave Presoclave III 80L, JP Selecta) at 121 ºC for 21 minutes. After sterilisation, the media were supplemented with concentrations of DESs from 0 (control) to 450 mM. The possible synergistic effect in the DES was also evaluated by adding the individual components separately. The concentrations of the HBA and HBD used correspond to those contained in the DES (HBD is two times higher). Both DES and individual components were sterilised by UV for 30 minutes and then added to the sterilised medium. The incubation experiments were conducted in 500 mL Erlenmeyers containing 100 mL of complex medium and inoculated with 1% (v/v) of *H. mediterranei* cells. This pre-inoculum was grown in the same complex medium containing 25% of inorganic salts solution and 0.5% (w/v) yeast extract, buffered with 100 mM of MOPS. Cells were grown at 42ºC and constant shaking at 170 rpm (Multitron Standard, Infors HT). Pre-inoculum was obtained in the exponential phase to obtain more metabolically active cells. Growth conditions also included 42 ºC and constant shaking at 170 rpm. Haloarchaeon growth was monitored by measuring the absorbance at 600 nm (Cary 60 UV-Visible Spectrophotometer, Agilent Technologies). To obtain a well-defined growth curve, all studies were performed with 6 replicates. Growth rate (μ) and doubling time (d.t) were calculated according using the equations: **μ= ln(X-X0)/(t-t0)**, where X and Xo represent the absorbance value at the end and the start of the exponential phase (respectively), and t and t0 the time in this period.[4]

To monitor the growth using DESs and individual components as nitrogen and carbon sources (in connection with potential bioremediation capabilities), cells were grown in a defined minimal medium containing 25% (w/v) of inorganic salts as previously mentioned, 20% glucose, 15 mM NH_4Cl, 1 mM phosphate salts (sodium dihydrogen phosphate dihydrate/disodium hydrogen phosphate dodecahydrate) (Na_2HPO_4/NaH_2PO_4) and 0.005 g/L of ferric chloride ($FeCl_3$). In the cultures for the study of the potential consumption of DESs or individual components as nitrogen and carbon source, NH_4Cl or glucose was not added, respectively (DESs or individual components were added in substitution). DESs were tested in 3 different concentrations: 100 mM, 200 mM and 300 mM. HBAs were introduced in the same concentrations as DESs, while HBDs were introduced two times higher (200 mM, 400 mM and 600 mM). To monitor the growth, the absorbance value at 600 nm was measured during the culture. Cultures were carried out in triplicates.

Statistical analysis

Experiments regarding the toxicity test of different DESs and individual components were carried out with 6 replicates. Growth curves and growth rates are expressed as the mean and the standard deviation (SD). Prism GraphPad Prism version 8 for Windows (GraphPad Software, San Diego, CA, USA) was used to determine and represent this data. In the growth rates comparison, differences within and between concentrations and components were evaluated by one-way ANOVA followed by a multicomparison Tukey´s test.

pH measurements data

Table S2. pH measurements during first 96 hours of culture for all the DESs and individual components tested in complex media. pH measures were carried out using MU 6199L multi-parameter (pHenomenal, VWR).

Compound	Concentration	0 h	24 h	48 h	72 h	96 h
**Control culture**	**-----------------**	7.64 ± 0.02	7.73 ± 0.04	7.89 ± 0.02	7.87 ± 0.02	7.89 ± 0.03
**AcChCl: acetamide (DES 1)**	**100 mM**	7.37 ± 0.01	7.01 ± 0.04	7.10 ± 0.02	7.19 ± 0.05	7.44 ± 0.08
	**200 mM**	7.18 ± 0.02	6.69 ± 0.02	6.61 ± 0.04	6.72 ± 0.01	7.02 ± 0.08
	**300 mM**	7.14 ± 0.03	6.58 ± 0.02	6.39 ± 0.01	6.37 ± 0.03	6.39 ± 0.01
	**350 mM**	7.34 ± 0.01	6.70 ± 0.02	6.44 ± 0.01	6.31 ± 0.02	6.31 ± 0.03
	**400 mM**	7.28 ± 0.01	6.67 ± 0.02	6.28 ± 0.03	6.06 ± 0.04	5.87 ± 0.087
	**450 mM**	7.28 ± 0.01	6.66 ± 0.02	6.12 ± 0.03	5.85 ± 0.03	5.77 ± 0.03
**ChCl: acetamide (DES 2)**	**100 mM**	7.34 ± 0.00	7.33 ± 0.01	7.45 ± 0.02	7.55 ± 0.01	7.72 ± 0.01
	**200 mM**	7.35 ± 0.00	7.35 ± 0.02	7.41 ± 0.02	7.67 ± 0.01	7.72 ± 0.00
	**300 mM**	7.37 ± 0.01	7.15 ± 0.03	7.33 ± 0.01	7.43 ± 0.02	7.59 ± 0.01
	**350 mM**	7.35 ± 0.01	7.36 ± 0.01	7.40 ± 0.01	7.41 ± 0.01	7.43 ± 0.02
	**400 mM**	7.33 ± 0.00	7.35 ± 0.01	7.35 ± 0.02	7.33 ± 0.00	7.37 ± 0.01
	**450 mM**	7.35 ± 0.00	7.36 ± 0.02	7.43 ± 0.03	7.54 ± 0.06	7.60 ± 0.00
**AcChCl: urea (DES 3)**	**100 mM**	7.59 ± 0.04	7.13 ± 0.01	7.20 ± 0.01	7.26 ± 0.02	7.31± 0.01
	**200 mM**	7.65 ± 0.05	6.92 ± 0.04	6.92 ± 0.03	6.98 ± 0.01	7.15 ± 0.02

	**300 mM**	7.28 ± 0.02	6.54 ± 0.04	6.55 ± 0.03	6.33 ± 0.07	6.03 ± 0.02
	**350 mM**	7.26 ± 0.03	6.56 ± 0.02	6.38 ± 0.022	6.01 ± 0.01	5.79 ± 0.07
	**400 mM**	7.27 ± 0.01	6.36 ± 0.02	5.99 ± 0.05	5.63 ± 0.05	5.50 ± 0.03
	**450 mM**	7.31 ± 0.02	6.22 ± 0.03	5.88 ± 0.01	5.54 ± 0.07	5.34 ± 0.12
**ChCl: ethylene glycol (DES 4)**	**100 mM**	7.35 ± 0.00	7.36 ± 0.01	7.40 ± 0.03	7.43 ± 0.02	7.57 ± 0.01
	**200 mM**	7.30 ± 0.00	7.32 ± 0.01	7.36 ± 0.02	7.40 ± 0.01	7.47 ± 0.03
	**300 mM**	7.23 ± 0.01	7.31 ± 0.00	7.32 ± 0.03	7.40 ± 0.02	7.49 ± 0.01
	**350 mM**	7.32 ± 0.00	7.34 ± 0.05	7.40 ± 0.02	7.44 ± 0.03	7.50 ± 0.03
	**400 mM**	7.30 ± 0.01	7.39 ± 0.03	7.40 ± 0.03	7.48 ± 0.02	7.51 ± 0.04
	**450 mM**	7.29 ± 0.01	7.30 ± 0.03	7.34 ± 0.01	7.39 ± 0.05	7.46 ± 0.04
**AcChCl**	**100 mM**	7.56 ± 0.00	7.12 ± 0.02	7.31 ± 0.03	7.38 ± 0.00	7.45 ± 0.01
	**200 mM**	7.68 ± 0.02	6.73 ± 0.02	6.83 ± 0.010	7.05 ± 0.01	7.22 ± 0.03
	**300 mM**	7.48 ± 0.01	6.43 ± 0.04	6.51 ± 0.03	6.58 ± 0.09	6.93 ± 0.02
	**350 mM**	7.39 ± 0.01	5.99 ± 0.06	6.31 ± 0.01	6.68 ± 0.02	6.86 ± 0.02
	**400 mM**	7.14 ± 0.03	5.50 ± 0.09	6.26 ± 0.02	6.55 ± 0.01	6.63 ± 0.05
	**450 mM**	7.52 ± 0.02	5.69 ± 0.02	5.93 ± 0.05	6.21 ± 0.04	6.45 ± 0.02
**ChCl**	**100 mM**	7.35 ± 0.01	7.34 ± 0.00	7.47 ± 0.03	7.62 ± 0.088	7.71 ± 0.01
	**200 mM**	7.40 ± 0.00	7.40 ± 0.08	7.50 ± 0.01	7.52 ± 0.01	7.67 ± 0.03
	**300 mM**	7.39 ± 0.00	7.39 ± 0.00	7.54 ± 0.00	7.59 ± 0.03	7.68 ± 0.01
	**350 mM**	7.47 ± 0.01	7.52 ± 0.00	7.55 ± 0.02	7.56 ± 0.02	7.57 ± 0.01
	**400 mM**	7.32 ± 0.00	7.35 ± 0.01	7.35 ± 0.01	7.33 ± 0.01	7.36 ± 0.01
	**450 mM**	7.35 ± 0.01	7.40 ± 0.01	7.45 ± 0.00	7.51 ± 0.01	7.59 ± 0.08
**Acetamide**	**200 mM**	7.63 ± 0.02	7.57 ± 0.01	7.51 ± 0.00	7.69 ± 0.02	7.73 ± 0.01
	**400 mM**	7.66 ± 0.01	7.65 ± 0.02	7.57 ± 0.02	7.66 ± 0.00	7.69 ± 0.01
	**600 mM**	7.60 ± 0.01	7.58 ± 0.01	7.65 ± 0.02	7.73 ± 0.022	7.73 ± 0.02
	**700 mM**	7.52 ± 0.01	7.53 ± 0.00	7.61 ± 0.02	7.70 ± 0.01	7.78 ± 0.02
	**800 mM**	7.50 ± 0.01	7.48 ± 0.01	7.62 ± 0.06	7.77± 0.00	7.80 ± 0.00
	**900 mM**	7.50 ± 0.01	7.49 ± 0.01	7.43 ± 0.01	7.47 ± 0.01	7.53 ± 0.01

**Urea**	**200 mM**	7.35 ± 0.05	7.41 ± 0.02	7.54 ± 0.01	7.63 ± 0.03	7.62 ± 0.01
	**400 mM**	7.47 ± 0.01	7.44 ± 0.05	7.54 ± 0.00	7.56 ± 0.02	7.61 ± 0.01
	**600 mM**	7.39 ± 0.02	7.46 ± 0.04	7.51 ± 0.01	7.55 ± 0.01	7.58 ± 0.01
	**700 mM**	7.53 ± 0.01	7.42 ± 0.04	7.52 ± 0.01	7.55 ± 0.01	7.61 ± 0.00
	**800 mM**	7.37 ± 0.03	7.38 ± 0.00	7.56 ± 0.01	7.70 ± 0.02	7.79 ± 0.01
	**900 mM**	7.43 ± 0.00	7.45 ± 0.01	7.54 ± 0.03	7.60 ± 0.02	7.70 ± 0.01
**Ethylene glycol**	**200 mM**	7.33 ± 0.03	7.39 ± 0.02	7.41 ± 0.01	7.48 ± 0.02	7.52 ± 0.03
	**400 mM**	7.32 ± 0.01	7.34 ± 0.00	7.43 ± 0.03	7.49 ± 0.02	7.57 ± 0.03
	**600 mM**	7.31 ± 0.01	7.39 ± 0.02	7.40 ± 0.04	7.50 ± 0.03	7.60 ± 0.04
	**700 mM**	7.32 ± 0.02	7.35 ± 0.02	7.46 ± 0.01	7.52 ± 0.01	7.62 ± 0.02
	**800 mM**	7.30 ± 0.02	7.32 ± 0.02	7.40 ± 0.01	7.50 ± 0.02	7.53 ± 0.04
	**900 mM**	7.31 ± 0.03	7.36 ± 0.04	7.37 ± 0.01	7.42 ± 0.03	7.50 ± 0.01

References

[1] X. Marset, J. Torregrosa-Crespo, R. M. Martínez-Espinosa, G. Guillena, D. J. Ramón, Multicomponent synthesis of sulfonamides from triarylbismuthines, nitro compounds and sodium metabisulfite in deep eutectic solvents, *Green Chem.* **2019**, *21*, 4127–4132.

[2] B.-Y. Zhao, P. Xu, F.-X. Yang, H. Wu, M.-H. Zong, W.-Y. Lou, Biocompatible Deep Eutectic Solvents Based on Choline Chloride: Characterization and Application to the Extraction of Rutin from Sophora japonica, *ACS Sustain. Chem. Eng.* **2015**, *3*, 2746–2755.

[3] A. P. Abbott, G. Capper, S. Gray, Design of Improved Deep Eutectic Solvents Using Hole Theory, *ChemPhysChem* **2006**, *7*, 803–806.

[4] M. Giani, L. Gervasi, M. R. Loizzo, R. M. Martínez-Espinosa, Carbon Source Influences Antioxidant, Antiglycemic, and Antilipidemic Activities of Haloferax mediterranei Carotenoid Extracts, *Mar. Drugs* **2022**, *20*, 659.
